# Hand and Eye Dominance in Sport: Are Cricket Batters Taught to Bat Back-to-Front?

**DOI:** 10.1007/s40279-016-0516-y

**Published:** 2016-03-18

**Authors:** David L. Mann, Oliver R. Runswick, Peter M. Allen

**Affiliations:** 1 Department of Human Movement Sciences, Faculty of Behavioural and Movement Sciences, MOVE Research Institute Amsterdam, Vrije Universiteit Amsterdam, Van der Boechorststraat 9, 1081 BT Amsterdam, The Netherlands; 2 School of Sport, Health and Applied Science, St Mary’s University, Twickenham, London, UK; 3 Department of Vision and Hearing Sciences and Vision and Eye Research Unit, Anglia Ruskin University, Cambridge, UK

## Abstract

**Background:**

When first learning to bimanually use a tool to hit a target (e.g., when chopping wood or hitting a golf ball), most people assume a stance that is dictated by their dominant hand. By convention, this means that a ‘right-handed’ or ‘left-handed’ stance that places the dominant hand closer to the striking end of the tool is adopted in many sports.

**Objective:**

The aim of this study was to investigate whether the conventional stance used for bimanual hitting provides the best chance of developing expertise in that task.

**Methods:**

Our study included 43 professional (international/first-class) and 93 inexperienced (<5 years’ experience) cricket batsmen. We determined their batting stance (plus hand and eye dominance) to compare the proportion of batters who adopted a reversed stance when batting (that is, the opposite stance to that expected based on their handedness).

**Results:**

We found that cricket batsmen who adopted a reversed stance had a stunning advantage, with professional batsmen 7.1 times more likely to adopt a reversed stance than inexperienced batsmen, independent of whether they batted right or left handed or the position of their dominant eye.

**Conclusion:**

Findings imply that batsmen who adopt a conventional stance may inadvertently be batting ‘back-to-front’ and have a significant disadvantage in the game. Moreover, the results may generalize more widely, bringing into question the way in which other bimanual sporting actions are taught and performed.

## Key Points

**Table:** 

Cricket batsmen have a surprising advantage if they adopt the stance opposite to that expected based on their handedness (i.e., if right handers bat left handed and vice versa).
The advantage appears to be grounded in positioning the dominant hand further from (rather than closer to) the striking end of the bat.
Findings suggest that cricket batsmen may inadvertently be taught to bat ‘back-to-front’.

## Background

**Fig. 1:**
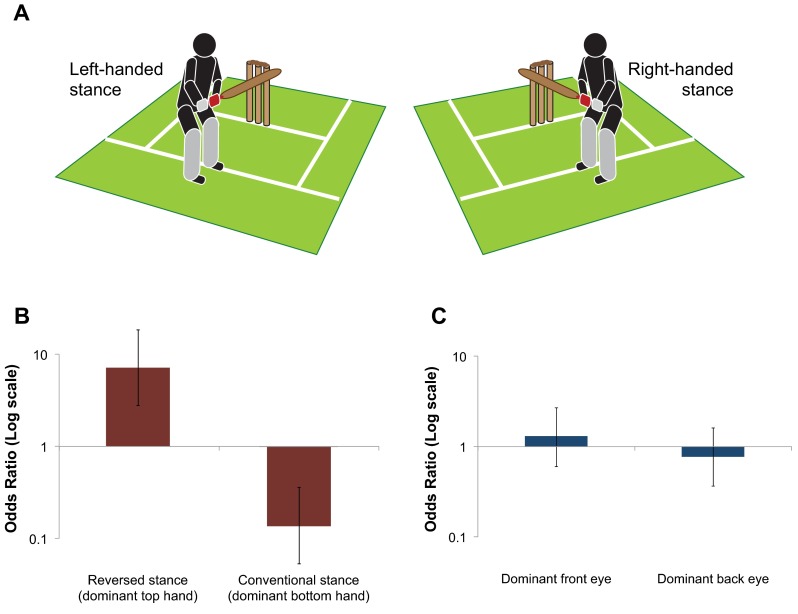
Cricket batting stances and their influence on achieving expertise. **a** Demonstration of conventional left-handed (*left*) and right-handed (*right*) batting stances. When adopting this conventional stance, the dominant hand (shown in *darker shading/red*) is placed lower on the handle so it is closer to the striking end of the bat. When adopting the reversed stance, a person who is right-hand dominant will adopt a left-handed stance, and a person who is left-hand dominant will adopt a right-handed stance. In the reversed stance, the hand placed further from the striking end of the bat (shown in *lighter shading/grey*) is the dominant hand. **b** Odds ratio comparing the proportion of professional and inexperienced batsmen who use a reversed or a conventional stance. *Error bars* show 95 % confidence intervals. **c** Odds ratio comparing the proportion of professional and inexperienced batsmen who bat with a dominant front or dominant back eye. *Error bars* show 95 % confidence intervals

Our hand dominance shapes the way we perform bimanual tasks. This is particularly the case when we use a tool (such as an axe or golf club) to strike a target. When doing so, we conventionally adopt a technique that places our dominant hand closer to the striking end of the tool. For instance, when playing cricket or baseball, we are usually taught to adopt a ‘right-handed’ or ‘left-handed’ stance that places our dominant hand closer to the striking end of the bat. Surprisingly, it is not clear why this is the case, and whether doing so provides the best chance of developing skill in that task. However, a small proportion of the population typically defies this convention and adopts the opposite stance to that which would be expected based on their handedness (which we call a *reversed* stance; see Fig. 1a for further explanation). Therefore, comparing the performance of those who adopt a conventional versus reversed stance provides an ideal opportunity to better understand which approach might best support the development of expertise.

The examination of skill in sporting activities provides some of the best evidence for the influence that handedness can have on the development of motor expertise. In particular, the highly competitive nature of elite sport ensures that small technical advantages can often afford significant competitive benefits. As a case in point, an over-representation of left-handers at the elite level of many sports is well-established [1, 2], particularly in interactive sports where players compete directly against opponents [2–4]. Brooks et al. [4] demonstrated that close to 50 % of the batsmen playing for the best-performing teams at the 2003 Cricket World Cup were left handed (compared with the 10–13 % predicted by population norms [5]), concluding that left-handed batsmen benefit from a *negative frequency-dependent effect* [2, 6, 7] because opponents are less accustomed to competing against left-handed batsmen and therefore are less adept at bowling to them (leading to poorer bowling strategies and accuracy). Crucially, this frequency-dependent effect would benefit anyone who bats using a left-handed stance, irrespective of their actual hand dominance.

Although there is a clear association between left handedness and success in sport [1, 2], this may be masking a more pervasive advantage afforded to some who play left handed. We have noticed that a surprisingly high proportion of seemingly left-handed batsmen in the sport of cricket adopt a reversed stance during competition; that is, they use a left-handed stance yet are actually right-hand dominant. For those familiar with cricket, left-handed batsmen such as Brian Lara, Chris Gayle, Clive Lloyd, David Warner, David Gower, Adam Gilchrist, Alistair Cook, Justin Langer, Michael Hussey, Mark Taylor, Kumar Sangakkara, and Matthew Hayden stand out as being some of the greatest batsmen of the modern era. What appears to have been largely overlooked (both by most people familiar with the game and by previous studies of handedness) is that, while each of these players bats left handed, they all bowl with their right hand (i.e., they bat using a reversed stance). If those players were to have benefitted from using a reversed stance, then we would expect to find they possess a specific advantage (*reversed-stance advantage hypothesis*) above and beyond the negative frequency-dependent effects available to all left handers. Moreover, a reversed-stance advantage should also be evident for those who are left-hand dominant yet bat right handed. In support, other modern-day greats such as Michael Clarke and Inzamam-ul-Haq bat right handed yet bowl with their left hand. Therefore, by adopting a reversed (right-handed) stance, they have foregone the potential frequency-dependent effects they would have benefitted from by batting left handed. Given the apparent wealth of high-quality players who adopt a reversed stance, it could be that doing so affords some sort of competitive advantage when batting that cannot be explained by a frequency-dependent effect. Crucially, if the reversed stance were to provide the best chance of developing skill in hitting, it would suggest that by teaching batsmen to use a conventional stance, coaches may be inadvertently teaching players to bat ‘back-to-front’ and could be harming rather than maximizing their chance of developing expertise.

There are two primary reasons to believe that a reversed stance might offer an advantage when batting. First, the reversed stance places the player’s dominant hand at the top rather than the bottom of the handle. This could confer technical advantages: in cricket batting, the top hand is typically responsible for controlling and guiding the path of the bat to hit the ball, so it may be an advantage for the hand with the greatest dexterity to perform those roles (dominant-hand explanation). Second, in tasks that require targeting people generally have a preference to rely on the visual input from one of the two eyes (the dominant eye), and the reversed stance increases the likelihood that the dominant eye is the ‘front’ eye in a side-on activity like batting (hand and eye dominance are matched in approximately 66 % of cases [8]). From our observations in both cricket and baseball, coaches and applied practitioners (e.g., optometrists) sometimes alter a batter’s stance to ensure that the dominant eye has a clear view of the ball. It could be that those with their dominant eye as the front eye are conferred an advantage because it ensures the dominant eye has an unobstructed view of the oncoming ball [9] (dominant-eye explanation).

The aim of this study was to determine whether a reversed stance provides an advantage in the development of expertise in a bimanual hitting task. We did so in the sport of cricket by testing the batting stance plus hand and eye dominance of 43 professional (international or first class) and 93 inexperienced cricket batsmen. We hypothesized that the reversed stance would offer a specific advantage in batting above and beyond that available to left handers as a result of frequency-dependent effects. We show that batsmen have a stunning advantage if they defy convention and adopt a reversed stance, and that the findings are supported by a significant over-representation of modern-day international batsmen who bat using a reversed stance. The results imply that most cricket batsmen are taught to bat ‘back-to-front’ and call into question the manner in which other bimanual motor actions are taught and performed.

## Methods

### Participants

A total of 43 professional male cricket players (mean age 29.6 years, standard deviation [SD] 5.6) and 93 inexperienced male cricketers (mean age 24.1 years, SD 7.2) participated in the study. The professional group were all members of a first-class and/or international cricket team: 26 had played at international level (ten had played test cricket) and 17 had played at first-class level. The professional players were all selected in their team based on their skill as a batsman (i.e., as a specialist batsman, a wicketkeeper/batsman, or as an all-rounder—someone who bats and bowls). Participants in the inexperienced group had less than five years’ cricket experience (mean 1.2 years, SD 1.4), with most not actively participating in organized cricket at the time of testing. The experimental procedure conformed to the ethical standards of the Declaration of Helsinki and was approved by the Ethics Committee of the Faculty of Human Movement Sciences at Vrije Universiteit Amsterdam. Participants were informed about the nature of the study and signed informed consent forms prior to testing.

### Procedure

We determined the hand dominance, eye dominance, and batting stance of all participants.

#### Hand Dominance

To determine hand dominance, participants completed the *Edinburgh Handedness Inventory—Short Form* [10]. This validated questionnaire provides a measure of handedness by testing the hand used during four activities of daily living: writing, throwing, using a toothbrush, and using a spoon. For each of the four activities, participants rated whether they use their right or left hand for that activity on a scale from one (always right) to five (always left). According to the questionnaire guidelines, participants whose average score across all four tasks was greater than three were classed as left-hand dominant, those whose score was below three were classed as right-hand dominant, and those with a score equal to three were classified as mixed dominance [10]. One professional and one inexperienced player had mixed hand dominance and were therefore excluded from all analyses. Handedness surveys were unavailable for five of the professional players. Consistent with previous studies [1], for those players we assumed the hand they used when bowling was their dominant hand (classifying one as reversed and four as conventional). In support, the hand used for bowling is almost always that used for throwing,^1^ and there was 96 % agreement (124/129 participants) between the dominant hand established by the questionnaire and that used for throwing.

#### Eye Dominance

**Fig. 2:**
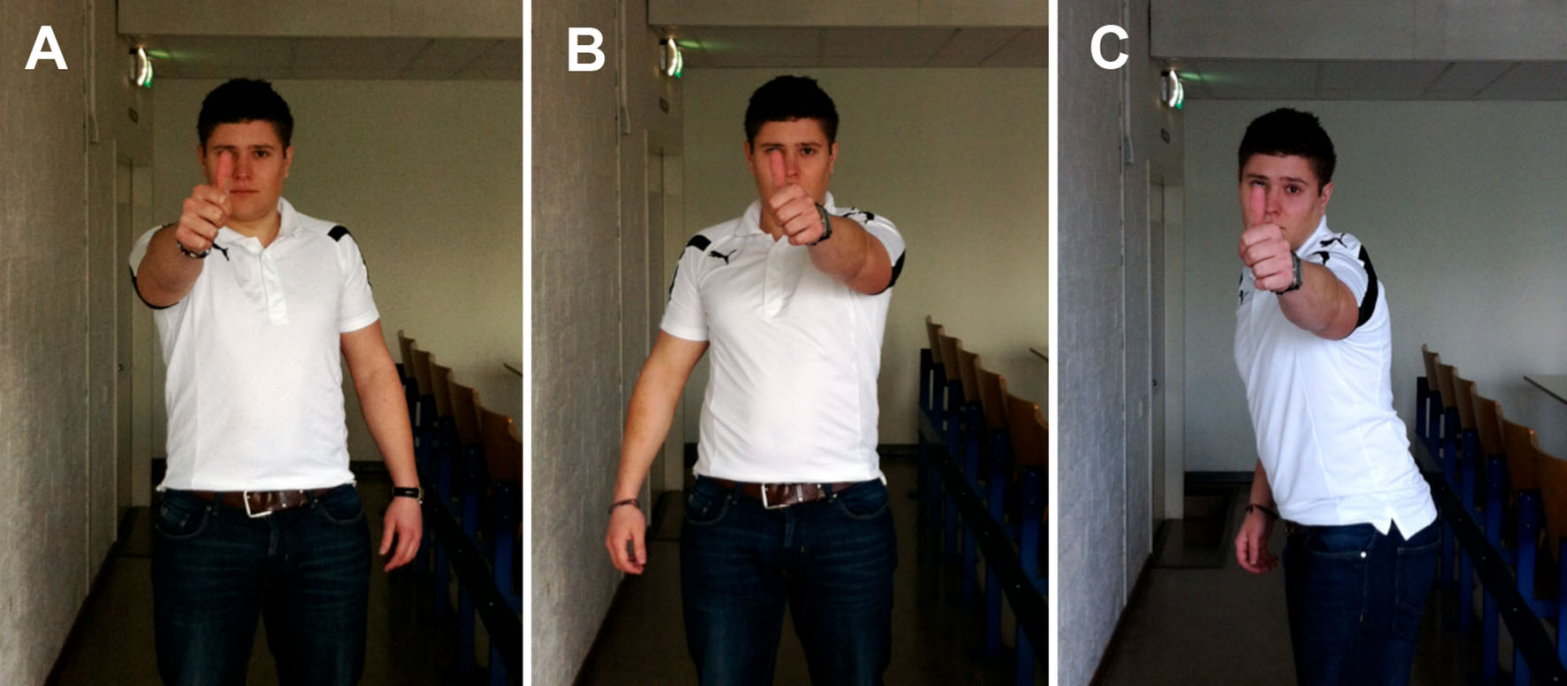
A photograph taken during each of the three tests of eye dominance. **a** The right-hand front-on test, **b** the left-hand front-on test, and **c** the batting eye dominance test. All three photographs show right eye dominance

Eye dominance can change depending on the conditions in which it is tested [11–13]. To account for this, three different tests of eye dominance were performed (Fig. 2). All three tests were based on a modified version of the Porta test [14], with a camera used to produce material evidence of eye dominance. For each test, participants stood three meters from a camera positioned at the participant’s eye level. Two of the three tests were performed using a front-on stance. In the *right-hand front-on test*, participants stood front-on to the camera, raised their right arm, and pointed directly at the center of the camera lens with both eyes open. When the participant confirmed that he was pointing at the center of the lens, a photograph was taken. This procedure was repeated for the *left-hand front-on test* when pointing with the left arm. The third test was one of *batting eye dominance*, where participants adopted their side-on batting stance and looked towards the camera. All inexperienced participants knew the stance they would typically adopt as they were from cricket-playing countries and had at some time played the game (formally or informally). From the batting stance, participants were asked to raise the arm nearest the camera and point towards the center of the lens with both eyes open. A photograph was taken when the participant confirmed he was ready.

Eye dominance was established by viewing the photographs of the participants. In each of the three tests, the dominant eye was determined by selecting the eye participants used to align themselves with the camera. If the finger/thumb was aligned with one of the eyes then that eye was deemed to be the dominant eye (e.g., if the right eye was in any way obscured by the finger/thumb, the participant was deemed to be right-eye dominant and vice versa). If the finger/thumb was placed between the two eyes rather than in any way obscuring an eye then the dominance was deemed to be mixed. The dominant eye established during the batting eye dominance test was expected to be the one most likely to be preferred during batting, so it was the eye used as the ‘dominant eye’ for further analyses. One of the professional players was found to have mixed eye dominance with this test; however, it was the same participant who had mixed handedness and had already been excluded from all analyses (the player was a relatively inexperienced and less accomplished all-rounder who had played only two first-class matches, and who bats and bowls left handed).

We checked the agreement between the three different tests of eye dominance. The agreement between the two front-on tests was 85 %, that between the batting eye dominance test and the right-hand front-on test was 87 %, and that between the batting and left-hand front-on test was 91 %. However, the best agreement was between the result for the test of batting eye dominance and that found during the corresponding front-on test using the same hand (95 %; i.e., if batting right handed, we compared the batting test with the left-hand front-on test and vice versa). This suggests it was the hand used during the test that caused most of the variability between the tests.

#### Batting Stance

Batting stance was determined on the basis of the stance that participants adopted in the test of batting eye dominance; a stance with the left foot closer to the camera was classified as a right-handed batting stance, and a stance with the right foot nearer the camera was classified as a left-handed batting stance.

### Additional Data

A preliminary check of the handedness data revealed very high agreement between the dominant hand determined by the questionnaire and the hand used when throwing (96 % agreement). Given that the throwing hand is generally also the hand used for bowling, we decided to use the bowling hand as a proxy for the dominant hand and to perform further analysis to check whether the proportion of professional batters who adopted a reversed stance in our sample was representative of that in the wider population of international-level batsmen. To do so, we collected additional data on the bowling hand and batting stance of (1) the 100 highest-ranked batsmen in the world, and (2) batsmen at the 2003 Cricket World Cup (matching the sample from the aforementioned study by Brooks et al. [4]). Data on the bowling hand and batting stance were collected from the match records of first-class and international cricket matches available on the website of ESPN Cricinfo (http://www.espncricinfo.com/ci/content/stats/index.html). If a player had not bowled in a match (typically wicketkeepers), we excluded that player from all analyses, as we had no way of determining their dominant hand.

#### Highest-Ranked International Batsmen

The International Cricket Council (ICC) ranks the 100 best-performing batsmen on an on-going basis for those who play in international tests, 1-day, and T20 matches (the three different formats of the game). We chose to use the rankings for the test batsmen, as this represents what is typically considered the most challenging form of the game. The rankings we used were accessed from the ICC website (http://www.icc-cricket.com/player-rankings/mens-test) on 15 November 2014. Data about the bowling hand were unavailable for seven batsmen, so only 93 batsmen were included in the final analysis.

#### Batsmen at the 2003 Cricket World Cup

Brooks et al. [4] reported, based on their analysis of batsmen taking part in the 2003 Cricket World Cup, that left-handed batsmen benefit from a negative frequency-dependent effect. We re-analyzed these data to determine whether the benefits experienced by the left-handed batsmen were better explained by an advantage for those who adopt a reversed stance. Of the 205 players reported to have batted at the 2003 World Cup (data retrieved from http://www.espncricinfo.com/ci/content/stats/index.html), we excluded those for whom we could not establish the bowling hand (*n* = 12). For consistency with the data from Brooks et al. [4], we excluded those who had not been dismissed at least once in the group matches (*n* = 33). As a result, a total of 160 batsmen were included in our analysis.

### Statistical Analyses

Chi-squared testing was used to establish whether proportions (e.g., proportion of batsmen who adopted a reversed stance) differed across two groups. Odds ratios (ORs) were used to calculate the size effects using Eq. (1):

1 $$ {\text{OR}} = \frac{{\left( n \right){\text{Exposed cases }} \times \left( n \right){\text{Unexposed noncases}}}}{{\left( n \right){\text{Exposed noncases}} \times \left( n \right){\text{Unexposed cases}}}} $$

where, for example, when interested in the proportion of professional batsmen who bat using a reversed stance (when compared with the proportion of the inexperienced group): (*n*)Exposed cases = number of professional batsmen who bat with a reversed stance, (*n*)Exposed non-cases = number of inexperienced batsmen who bat with a reversed stance, (*n*)Unexposed cases = number of professional batsmen who bat with a conventional stance, (*n*)Unexposed non-cases = number of inexperienced batsmen who bat with a conventional stance.

In two cases, we pooled the players in our professional group with the 100 highest-ranked international batsmen and the players in the 2003 Cricket World Cup. Eight batsmen were in at least two of the three pooled groups, so their data were included only once. The data for the pooled group were then compared with those of the participants in our inexperienced group. To compare the advantages afforded to professional left-handed batsmen who used a conventional or reversed stance to those in the inexperienced group, we used a goodness-of-fit test because the number of inexperienced batsmen who did so was very low (three and two respective participants) and this would have violated the assumptions of a normal chi-squared test (needing a minimum of five observations in each cell of the contingency table). ORs were reported as a measure of effect size for the goodness-of-fit test (comparing the observed and expected frequencies).

We calculated 95 % confidence intervals (CIs) for each of the ORs using Eq. (2). Results were considered significant to *p* < 0.05 if the CI did not pass through the null value of one.

2 $$ 95 \,\, \%   \,\,{\text{CI}} = e^{{\left( {\ln \left( {\text{OR}} \right) \pm 1.96\sqrt {\frac{1}{a} + \frac{1}{b} + \frac{1}{c} + \frac{1}{d}}  } \right)}} $$

where *a*, *b*, *c*, and *d*, respectively, refer to the number of exposed cases, exposed non-cases, unexposed cases, and unexposed non-cases.

## Results

### Reversed Versus Conventional Stance

Adopting a reversed stance appears to offer a very significant advantage in becoming a professional batsman. Our group of professional batsmen were 7.1 times more likely to adopt a reversed stance than the inexperienced batsmen (40 % of the professionals vs. 9 % of the inexperienced batsmen; *χ*^2^(1) = 19.2, *p* < 0.0001; OR 7.1, 95 % CI 2.8–18.5; Fig. 1b).

The results strongly supported the dominant-hand explanation as the reason for the over-representation of professional batsmen who adopt a reversed stance. Placing the dominant eye at the front of the stance (closer to the bowler) did not change the likelihood of being in the professional group (43 vs. 38 %; *χ*^2^(1) = 0.28, *p* = 0.60; OR 1.2, 95 % CI 0.6–2.6; Fig. 1c) [9], whereas placing the hand at the top of the bat clearly did (40 vs. 9 %; OR 7.1, see previous paragraph). None of the conclusions changed if we used the results of the front-on tests rather than the batting eye-dominance test: placing the dominant eye at the front of the stance did not change the likelihood of being in the professional group if the results from the right-hand front-on dominance test were used (*χ*^2^(1) = 2.38, *p* = 0.12; OR 1.8, 95 % CI 0.8–4.1) or if the results of the left-hand front-on test were used (*χ*^2^(1) = 0.12, *p* = 0.73; OR 1.2, 95 % CI 0.5–2.6).

A small proportion of the population have inconsistent handedness [15], meaning that they write with one hand yet throw with the other (≈28.8 % of left-handed and 1.6 % of right-handed writers) [16]. It could be reasonable to hypothesize that the switched-stance batsmen are those who display inconsistency, as they would be more adept at using their non-dominant hand. However, this was not the case. We analysed the data for our participants for whom we had conclusive questionnaire data on the writing and throwing hands (*n* = 129) and found that only five had inconsistent handedness: four from the professional group (all reversed stance) and only one from the inexperienced group (conventional stance). As a result, the majority of our reversed-stance professional batsmen (75 %; 12 of 16 batsmen) did not have inconsistent handedness. Indeed, the significant advantage for the reversed-stance batsmen remained even when considering only those known to have consistent handedness (36 vs. 9 %; *χ*^2^(1) = 13.6, *p* < 0.001; OR 5.9, 95 % CI 2.1–16.4). Although the reversed-stance advantage cannot be explained by a particular benefit for those who have inconsistent handedness, the higher proportion in the professional group is suggestive of inconsistent handedness playing a role in the development of skill in batting.

To confirm that the advantage for reversed-stance batsmen generalized more widely to other elite batsmen, we examined the proportion of the 100 highest-ranked modern-day international cricket batsmen who adopt a reversed stance (assuming the bowling hand as the dominant hand [1]). Almost one-third of the world’s best batsmen defy convention and adopt a reversed stance (30 %; *χ*^2^(1) = 13.5, *p* < 0.001; OR 4.5, 95 % CI 1.9–10.6; using the inexperienced group as controls). This is striking given our finding that only a small proportion of our control population of inexperienced batsmen (≈9 %) adopt a reversed stance when batting.

### Left-Handed Advantage

Consistent with previous studies [1, 4], our sample included a significant over-representation of professional batsmen who batted using a left-handed stance (14 of 42 batsmen; proportion of professional vs. inexperienced batsmen = 33 vs. 5 %; *χ*^2^(1) = 18.4, *p* < 0.0001; OR 8.7, 95 % CI 2.9–26.3). However, the results of the handedness questionnaires show that 13 of those 14 professional batsmen (93 %) were actually right-hand dominant. This provides some support for the idea that the advantage conferred on left-handed batsmen is largely for those who adopt a reversed stance (i.e., for batsmen who are right-hand dominant but bat left handed) rather than there being a more general frequency-dependent benefit for all who bat left handed (irrespective of whether they are right- or left-hand dominant). To check this, we re-examined the data from Brooks et al. [4] on batsmen at the 2003 World Cup and found that the majority of their left-handed batsmen (25/41; 61 %) were in fact right-hand dominant (based on their bowling hand). Moreover, for the 100 highest-ranked modern-day test batsmen, 70 % of those who bat left-handed are right-hand dominant (26/37).^2^ Overall, batsmen who are left-hand dominant and bat left handed (i.e., use a conventional stance) have an advantage in becoming a professional (9.8 % of professionals vs. 3.3 % of inexperienced; *χ*^2^(1) = 38.4, *p* < 0.00001, OR 3.2, 95 % CI 1.5–6.9; pooling the professional, 2003 World Cup, and 100 highest-ranked batsmen and comparing it with the proportion of our inexperienced batsmen), consistent with the idea that left handers benefit from a frequency-dependent effect. However, this benefit is strongly outweighed by the significantly greater advantage afforded to batsmen who are right-hand dominant but bat left handed using a reversed stance (21.3 % of professionals vs. 2.2 % of inexperienced; *χ*^2^(1) = 491.3, *p* < 0.00001, OR 12.1, 95 % CI 5.2–28.2; OR is outside of the 95 % CI for the left-handed batsmen who are left-hand dominant), demonstrating a significant reversed-stance advantage above and beyond that possible from a more general frequency-dependent effect.

Importantly, the reversed-stance advantage may not be exclusive to batsmen who use a left-handed stance, as the effect appears to generalize more widely to those who are left-hand dominant but bat using a right-handed stance. If there were to be a selective benefit for left-handed batsmen, the proportion of reversed-stance batsmen who bat right handed (but are left-hand dominant) should be lower than 10–13 % (the proportion of the population who are left-hand dominant [5]). Of our professional batsmen, 24 % of the reversed-stance batsmen batted right handed (4/17). To see if this finding applied more widely, we again pooled those data with the 100 highest-ranked and 2003 World Cup batsmen. We found the proportion of reversed-stance batsmen batting right handed (13/74; 18 % of reversed-stance batsmen) did not differ from the 10–13 % expected by chance (*χ*^2^(1) = 2.7, *p* = 0.10; OR 1.6, 95 % CI 0.6–4.2). Although a much larger sample would be necessary to conclusively demonstrate a reversed stance advantage when adopting a right-handed stance, the evidence at hand leads us to believe that the reversed-stance advantage is not exclusively conferred to left-handed batsmen, but may be apparent for both left-handed and right-handed batsmen.

## Discussion

The aim of this study was to determine whether a ‘reversed’ stance provides a significant advantage in the development of expertise in a bimanual sporting task. We tested the batting stance plus hand and eye dominance of professional and inexperienced cricket batsmen. If there were an over-representation of professional batsmen who adopt a reversed stance, this would provide evidence that a reversed stance is a better technique to use for batting. Consistent with our reversed-stance advantage hypothesis, we found that the professional batsmen were seven times more likely to adopt a reversed stance than the inexperienced batsmen. The over-representation could not be explained by a frequency-dependent advantage for left-handed batsmen, by the position of the dominant eye in the stance, or on the basis of inconsistent handedness; rather, the results appear to be grounded in the positioning of the dominant hand at the top of the bat handle. The findings indicate that a reversed stance provides a remarkable advantage in becoming a professional batsman and raise interesting questions about whether the findings would apply more widely to other bimanual tasks.

Given that the ‘conventional’ way of holding a cricket bat (with the dominant hand on the bottom of the handle) has remained basically unchanged since the invention of the game, we sought to discover how this convention first came about. To uncover this we visited the library at Lords Cricket Ground in London and found that the conventional stance used for cricket batting may have been modelled on the stance used for other bimanual hitting tasks. For instance, the first Marylebone Cricket Club (MCC) coaching manual published in 1952 [17] instructs batters to pick up a bat in the same manner they would pick up an axe. This too would typically ensure that the dominant hand is on the bottom of the handle and may explain why batters were originally taught to adopt such a grip when batting.

The modelling of cricket batting on other bimanual hitting tasks leads us to ask two questions: (1) why might there be a general preference for placing the dominant hand closer to the hitting implement when performing these tasks, and (2) how could doing so prove to be a *disadvantage* in the development of skill? Learners undergo different stages of learning when acquiring a new motor skill [18, 19], and we hypothesize that the influence of the position of the dominant hand in bimanual hitting might differ across the different stages of learning. More specifically, a conventional stance may provide a short-term advantage when first learning to use the tool yet be a disadvantage in the longer-term development of expertise. Consider a young child learning to use a hammer. At first, they will place their hand very close to the hitting end of the hammer to increase control (by decreasing the moment arm [20]) and thereby improve their hitting accuracy. However, as the child becomes more proficient in using the hammer, they typically move their hand further from the hitting end, effectively increasing the moment of inertia (the hand/pivot is further from the hitting end of the implement [20]) to enhance the power with which they can hit the target. Similarly for cricket batting, we hypothesize that the conventional stance may be beneficial when first learning because the dominant hand is lower on the bat, effectively increasing control and accuracy in the initial stages of learning. However, as cricket batsmen learn to refine their batting technique, they are taught to ensure their top hand is the one that provides most control when guiding the swing path of the bat. Therefore, in the longer term, it may be that those with a reversed stance enjoy the benefits of having their dominant hand (with greater dexterity) performing the bulk of the work required to swing and control the bat. It could be that the optimal learning approach would be to first learn using a conventional stance, but to switch to a reversed stance later in development. This hypothesis could be tested by (1) examining whether those who have become successful using a reversed stance did so when they first learned to bat or only later in development, and/or (2) designing training interventions that evaluate the efficacy of learning when adopting the reversed stance at the start of, or only later in, development.

In addition to the potential technical advantages conferred to reversed-stance batsmen while batting, they may also benefit from a bias in talent selection during development. Coaches and talent scouts generally prefer young batsmen to possess an ideal ‘technique’ when batting, and a dominant top hand is likely to be associated with a more favorable technique when batting. Specifically, batsmen are generally expected to maintain a ‘straight bat’ when attempting to hit the ball so that the plane of the bat swing matches the oncoming ball (thus maximizing the margin for error at bat–ball contact). This is more likely to occur when the bat is predominantly controlled by the top hand [21]. If having the dominant hand at the top of the bat does allow for a better technique, then reversed-stance batsmen may benefit from an increased likelihood of being selected into representative teams and squads, offering better training and exposure to higher-level competition. These claims are clearly testable: kinematic analyses can be performed to test whether a dominant top hand does afford technical advantages when swinging the bat, and the selections made by coaches or scouts can be tested to see whether they have an unintentional bias towards selecting batsmen who bat using a reversed stance.

Given the popularity of the game of cricket, it is surprising that the advantage afforded by the reversed stance has not already been established. The majority of batsmen who adopt a reversed stance do so while batting left handed; therefore, the effect may have been largely masked by the more widely known over-representation of left-handed batsmen at the professional level [1, 4]. In contrast, the proportion of professional batsmen who are left-hand dominant yet bat right-handed is not high (≈4.5 % from our data). Therefore, it is not surprising that any over-representation of those batters has not stood out relative to the proportion of those found in the wider population, even though some of the best batsmen in the modern era have batted right handed yet bowled with their left hand (e.g., Michael Clarke and Inzamam-ul-Haq).

Although our results could not be explained on the basis of an advantage afforded by inconsistent handedness (writing and throwing with the opposite hand), the surprising number of our professional batsmen who did so (*n* = 4) suggests this may be a topic worthy of further investigation. In support, some of the best players of the modern era are known to have inconsistent handedness (e.g., Kane Williamson, Shane Watson, and Mitchell Johnson), and even Sachin Tendulkar, probably the best batsman in the last 70 years of international cricket, batted and bowled right handed yet writes with his left hand. It is possible that those with inconsistent handedness are less lateralized and may therefore benefit in a bimanual task such as batting. Alternatively, it could be that players with inconsistent handedness are simply more likely to adopt a reversed stance because they choose their batting stance on the assumption that it should match their bowling/throwing hand rather than the hand they write with.

Cricket batting represents one of many bimanual tasks where tools are used to strike targets. We have limited our examination to cricket, but the results may apply more widely. In golf, three of the four men to have won a major championship playing left handed were right-hand dominant. Similarly, some of the world’s best golfers (e.g., Ben Hogan, Arnold Palmer, Nick Price) were left-hand dominant but played right handed [22]. Furthermore, almost half of the top ten presently active batters playing Major League baseball (as measured by batting average) bat left handed yet throw (and write) right handed (four of nine batsmen; the tenth bats both right and left handed).^3^ These observations suggest the reversed stance benefits apparent in cricket batting may apply more widely to the performance of other tasks, particularly those in which performers hit a target with power to maximize success. It could be that a dominant top hand is beneficial in those tasks (e.g., cricket and baseball batting, driving in golf), whereas a dominant bottom hand could be best when prioritizing precision (e.g., golf putting and dribbling in field hockey). A comparison of the technique used by skilled and less-skilled performers of those tasks would help uncover the optimal means of performing each task.

It is not clear why those who adopt a reversed stance might have chosen to do so, though in many cases it appears to have happened by chance. One of Australia’s best ever cricket batsmen, Michael Hussey, is right-hand dominant but learned to bat left handed to emulate his childhood idol, Allan Border [23]. American golfer Phil Mickleson, a five-time major-championship winner, is right handed but learned to play left handed to mirror his father’s right-handed swing [22]. Chance occurrences like these may have bestowed an unexpected advantage on those who inadvertently adopted a reversed stance. And the results suggest, at least in cricket, that by adopting the conventional stance, batsmen may have been unintentionally taught to bat ‘back-to-front’ and might not have maximized their potential in the game.

## Footnotes

1 We are not aware of any cases of cricketers who throw with one hand yet bowl with the other.

2 We expect that the higher proportion of reversed-stance left-handed batsmen in our sample is a result of chance, although participants in our sample were largely playing in the English first-class competition, and therefore may not provide as even a representation of the international playing community as our two additional groups. A country- or cultural-specific bias could underpin the difference.

3 We sourced the list of top ten currently active batsmen, along with their playing stance and throwing arm, from the official MLB website (http://www.mlb.com; retrieved 30 Jan 2016). We searched Google for images of each player signing autographs to establish the writing hand (e.g., “Ichiro Suzuki signing autographs”). All ten batsmen throw right handed: four bat left handed, five right handed, and one both right and left handed.
